# Do patients and the public understand and feel engaged and valued after reading outcome set study invitations? A UK qualitative study

**DOI:** 10.1136/bmjopen-2025-111016

**Published:** 2026-08-18

**Authors:** Heather Barrington, Rebecca Craven, Paula R Williamson, Bridget Young

**Affiliations:** 1 Health Data Science Department, University of Liverpool, Liverpool, UK

**Keywords:** STATISTICS & RESEARCH METHODS, QUALITATIVE RESEARCH, Patient Participation, Research Design

## Abstract

**Abstract:**

**Background:**

The use of core outcome sets (COSs) in health research is widely recommended. COSs are developed with input from key decision makers, patients being a vital group to ensure COS relevance. However, recruitment to COS studies can be challenging.

**Objectives:**

The study aimed to explore what patients and the public think of COS study invitations, which are usually the first document a patient reads about a COS study.

**Design:**

Qualitative study involving focus groups and interviews. Analysis of transcribed data drew on reflexive thematic analysis.

**Participants and setting:**

Focus groups and interviews were conducted with a diverse range of patients and the public (n=31) from community and patient groups in North West England, exploring their perceptions of a sample of COS study invitations. Participants were eligible if aged over 18 years, they could speak English and had never previously participated in a COS study.

**Results:**

Themes identified included understanding, engagement, safety and accessibility. Participants were concerned about excessive information in the invitations which made them confused. Fear of cybercrime caused numerous concerns and participants were reluctant to click on links or attachments. Trust in the people sending the invitations was key. Participants wanted to see messages that COS studies had a meaningful purpose to feel their contributions were valuable. They were keen to contribute to improving future ‘treatment’ but less motivated to improve ‘research’. Messages that evoked a feeling of connection with others who they could relate to were positively regarded. Participants also highlighted ways that invitations could be made more accessible.

**Conclusion:**

COS invitations can present numerous challenges for potential participants, but our study indicates ways that COS developers can address these challenges and make their invitations more appealing to potential participants. As part of the wider Good Research Invitation Study the findings have been used to inform guidance on designing a good COS invitation

**Strengths and limitations of this study**

- This qualitative study, which involved focus groups and online interviews, explored what patients and the public think of core outcome set study invitations. To enhance credibility, previously used core outcome set study invitations were incorporated as stimulus material.
- A diverse range of participants including those from seldom heard groups participated, including those from socio-economically disadvantaged communities.
- There was limited ethnic representation, and the study was conducted in the UK; we cannot assume the findings are more widely transferable across ethnic groups and countries.
- Our study participants were willing research participants, as such their perceptions of research invitations may be more positive than the wider public.

## Introduction

 The selection and reporting of patient-important outcomes (what is measured in a study to assess if an intervention works) has been highlighted as an area for improvement in guidance to address a key challenge in evidence synthesis - heterogeneity in outcomes.^1^ Standardised outcomes for health conditions or interventions can address this challenge. In clinical trials, standardised outcomes have been termed ‘core outcome sets’ (COS) and are considered ‘the minimum that should be measured and reported in all clinical trials of a specific condition’.^2^ COS development requires the participation of key stakeholders, including patients, clinicians, researchers, funders and other key decision makers.^3^ In this article, the term ‘patients’ includes service users, carers and the public who contribute their lived experience to research.

Patient participation is important as clinicians alone may fail to prioritise patient-important outcomes. For example, a rheumatology COS initiative, Outcome Measures in Rheumatoid Arthritis Clinical Trials (OMERACT), historically had not included patients. However, later work by OMERACT exploring the patient perspective identified an important outcome—fatigue—which had not previously been included in their COS development with clinicians alone.^4–6^ Dodd *et al*^7^ reviewed a cohort of published COS studies finding that studies including the perspectives of patients were more likely to include life impact outcomes. Without patient input into COS, researchers may be unaware of the outcomes patients strive for when they embark on a treatment.

Most COS studies now include patients as participants.^8^ Consensus-building processes such as Delphi surveys are often used in COS development,^9^ but these processes can be difficult^10^ for patients and the public to take part in and both patients and COS developers have highlighted concerns that patients are often confused about the purpose of a COS.^11 12^ Challenges in recruiting patients and the public have also been reported^13–17^ and patients tend to be in the minority as stakeholders in COS studies.^13^ Over recent years there has been an increased emphasis on the importance of inclusivity in research,^18–21^ which was further emphasised by the COVID-19 crisis, with those populations most at risk of adverse outcomes often being underrepresented in COVID-19 research.^22 23^ However, we know little about the demographic characteristics of COS participants as this is rarely reported^13^ so it is unclear how inclusive these studies are.

If a COS is to be relevant to the patient population it is designed for, COS researchers need to ensure adequate patient participation and diversity among those who do take part. The recruitment approach is likely to be important. Most COS developers use remote methods to initially invite patient participants such as emails, social media posts and flyers in clinics.^13^ To our knowledge, no research has previously explored what patients and members of the public think of such research invitations. If patients are to be included in the process of prioritising outcomes and reflect the patient population the COS is designed for, we need to ensure that participant-facing resources used in COS studies are understandable, accessible and engaging. We designed the Good Research Invitation Study to first explore the perspectives of members of the public on study invitations as these are the first documents most potential participants see when invited to join a COS study. As subsequent participant-facing documents, such as participant information sheets, may not be read unless an initial invitation has first engaged a person’s interest, these subsequent documents were not the focus of our study. Our aim was to gauge the extent to which a sample of COS study invitations were engaging, understandable and made patients feel valued because these documents are a key first step in ensuring COS studies include a diversity of participants. Understanding this may help future COS developers design invitations that better address the information needs of patients and the public at the invitation stage of a COS study. A second aspect of the Good Research Invitation Study involved developing a list of recommendations based on the qualitative findings which we took to a consensus meeting to refine and finally agree guidance for COS developers (this study is reported separately).^24^

## Methods

### Design

We conducted a qualitative focus group and interview study (the study protocol is provided in online supplemental appendix 1). This article has been reported in accordance with the Standards for Reporting Qualitative Research (SPQR).^25^ We used focus groups as our main method of data collection as the subject matter was not overly sensitive, and to enable a range of opinions to be expressed and expanded on through group interaction.^26^ A pragmatic ontological approach enabled us to explore participants’ perceptions of a sample of COS invitations.

### Collation of stimulus materials

We used recent COS invitations as stimulus material for our focus groups to ensure authenticity. We accessed the invitations via COS study teams identified through the Core Outcome Measures in Effectiveness Trials (COMET) Initiative, which maintains a database of COS studies. COS developers who had recently completed recruitment or publication (published since January 2020) were invited to send us examples of their COS invitations. Eligible invitations were aimed at adult patients in the Delphi component of a COS study and written in English.

Three authors (HB, RC and BY) met to review 14 of over 60 collated invitations and select six for use as focus group stimulus material: two emails, two social media posts and two flyers. We selected what we considered to be the most accessible invitations, to provide opportunities for focus group participants to highlight favoured aspects of the invitations. Three reserve invitations were also identified in case these were needed. Finally, we prepared several additional key messages that COS developers had used or might use to encourage participation for discussion in the focus groups if time allowed. Selection of these was informed by Cialdini’s Principles of Persuasion^27^ (online supplemental appendix 2) that are believed to guide people’s decision making in a range of contexts. For example, Cialdini’s scarcity principle could be represented in a COS study invitation by a message that the study was a ‘rare’ opportunity to take part. Other messages were similarly identified for use (Topic guide online supplemental appendix 3). Identifying details of researchers were removed from the invitations prior to their use in the focus groups.

### Sampling and recruitment of participants

COS studies are conducted with a range of participants, including members of the public as well as patients with chronic or acute conditions. We therefore purposively sampled from community groups as well as patient support groups. We also sampled groups with the aim of recruiting a diverse sample in terms of socio-economic background, ethnicity, age and gender. For logistic reasons our sampling focused on groups in Northwest England.

Invitations were emailed to each group’s gatekeeper who disseminated the invitations to their members. The researcher (HB) also attended a regular meeting of each group to explain the study to potential participants. Participants interested in taking part were asked to complete a demographic characteristics form, with a plan to purposively sample individuals based on their demographic characteristics if we received more interest than we had places. Interviewees were provided with a £15 voucher for taking part.

### Data collection

Data were collected between September 2023 and March 2024. The face-to-face focus groups and online interviews, which were conducted by HB in English, were semi-structured and topic guided. The topic guide was co-produced by the authors and informed by piloting. Participants were provided with the sampled invitations and asked to consider how understandable and interesting an invitation was, and whether it made them feel valued. Where time allowed, we introduced some of our additional invitations or some of the key messages described above. Field notes were kept, informing the later analysis.

Analysis was ongoing throughout data collection, iteratively informing further development of the topic guide. Informed consent was obtained from all participants. The audio recordings of the focus groups and online interviews were transcribed by a professional transcription agency. Transcripts were checked and anonymised before being analysed.

### Data analysis

We drew on Braun and Clarke’s reflexive thematic analysis approach.^28 29^ Analysis was initially deductive, focused on the three main elements of the research aim: understanding, engagement and feeling valued, evolving to an inductive approach as analysis developed. HB led the main analysis, reading the transcripts multiple times, reflecting also on focus group field notes, then organising and coding the data into nodes assisted by QSR NVivo V.14 software. BY, PW and RC also read, interpreted and discussed with HB a sampled transcript and HB met regularly with BY to discuss and develop the analysis and categorisation of the data. BY, HB and PW agreed that data saturation had been achieved after six focus groups and two online interviews, involving 31 participants from diverse backgrounds.

Reflecting on the research team’s backgrounds, it is important to note that all are white middle-class females, but with varying professional roles. We acknowledge that our backgrounds and roles would likely influence our interpretation of the data and therefore outline them here. Specifically, HB and RC both had initial experience of the world of COS as members of the public and had previously participated as carers in COS studies. PW is the Chair of the international COMET Initiative and BY was the co-chair of COMET’s People and Patient Participation, Involvement and Engagement Working Group.

### Patient and public involvement statement

Patients and the public were involved as research partners in the study. One public research partner (with experience of participating in a COS study) collaborated on: the study design and participant-facing materials, transcript interpretation, production of an end-of-study infographic, dissemination of the findings and co-authoring this article. Four additional patient research partners (PRPs) were involved in piloting the data collection methods and two additional PRPs were involved in designing the infographic.

## Results

### Focus group recruitment and participants

Of 13 patient support and community groups contacted, 6 were recruited, comprising two patient support groups and four community groups. 40 potential participants completed initial expression of interest forms. Of these, 31 participated across six face-to-face focus groups and two online interviews. Focus groups lasted an average of 146 min (range 139–151 min), with the two interviews ranging between 65 and 83 min. Participants represented a diverse range of demographic and educational backgrounds: 17 were female, 27 were white, two were Asian/Asian British, one was of mixed or multiple ethnic groups and one was black European. Participants’ highest education levels were higher education (n=8), further education (n=9) and secondary education (n=14). The sociodemographic and age characteristics are presented in (table 1).

**Table 1:** Good Research Invitation Study participant characteristics

Participant	Age bracket	Index of Multiple Deprivation decile (lower value=more deprived)
1	55–64	1–3
2	55–64	1–3
3	35–44	1–3
4	65–74	1–3
5	65–74	1–3
6	75 and over	1–3
7	75 and over	1–3
8	75 and over	1–3
9	65–74	8–10
10	75 and over	8–10
11	65–74	4–7
12	25–34	8–10
13	45–54	4–7
14	55–64	8–10
15	75 and over	8–10
16	55–64	4–7
17	45–54	8–10
18	55–64	8–10
19	25–34	8–10
20	55–64	4–7
21	25–34	4–7
22	55–64	4–7
23	55–64	4–7
24	35–44	8–10
25	35–44	1–3
26	45–54	1–3
27	35–44	1–3
28	35–44	4–7
29	18–24	4–7
30	18–24	1–3
31	25–34	1–3

We present the findings structured into the themes of understanding (how understandable the invitation was), safety (including trust, cyber fear and data protection), engagement (including both motivating and demotivating factors) and accessibility (such as literacy and digital exclusion). Brief quotations from the data are provided in the following sections to illustrate findings, with additional quotations in online supplemental appendix 4 to further evidence the findings.

### Understanding

#### “You’ve got to get the words right!”

Communication with the public about health research has long been criticised for its complexity but the concerns raised by participants about the vocabulary in the invitations was more intense than we anticipated, particularly as we had aimed to sample invitations that were accessible in their messaging. Several participants were concerned about the use of what one participant called “big doctor words” (P3). Specific words that were notably problematic for many participants included ‘outcomes’, ‘core outcome set’ and ‘Delphi’. The word ‘outcome’ was often described as ‘jargon’ or ‘legal, clinical speak*’*. Indeed, one participant highlighted its abstract nature and the need for more definite language (online supplemental appendix 4, Quote1(Q1)). It was clear that the complexity of the vocabulary in invitations adversely impacted how participants processed information, even risking the researcher ‘losing’ a potential COS participant (Q2 and linking to the later theme of engagement in section 3.5).

Participants in one group emphasised that the invitations assumed patients knew the name of their diagnosis and noted that further explanation may be needed. Some invitations used terms such as ‘invaluable’ to emphasise the value of the patient’s perspective in the COS development process. However, as one participant highlighted, this adjective could be confusing or counterintuitive as the prefix ‘in’ is often associated with lacking something (such as the word invisible). Some confusion arose with the term ‘Principal Investigator’, with participants commenting that the term was suggestive of being scrutinised or having “done something wrong” (P18). Participants also highlighted the negative connotations associated with the term ‘critical outcomes’, linking it with serious illness and reinforcing the importance of choosing vocabulary sensitively.

Acronymisation was, as expected by the study team, problematic for many participants. Acronyms in invitations could be related to the health condition, to health, health research, patient organisations or in the abbreviated study title. However, most participants focused on the acronyms in short study titles that researchers had created from longer study titles. In devising short names for studies, researchers commonly use acronyms that represent or resemble words in common use. Often these take letters from the full titles of studies, which are generally long and complex. Sometimes researchers slightly change the usual spelling of the common word so that the letters contained in the acronym correspond to those in the long study title. While a small number of participants did not mind the acronyms, many were confused and distracted by them. For example, one participant struggled to get beyond reading what they felt was a misspelt study title:

P30: I spent more time on that than the entire poster ……. It made me question my own spelling ability.

Others highlighted that a seemingly misspelt acronym in the study title would make them question the abilities of the researchers, leading participants to mistrust the research. A participant in another group felt the study details should not even be included in the title, suggesting instead something as simple as *“*A new research study needs your help” (P14).

#### Sentence construction and presentation

Participants discussed the way that sentences were constructed and presented in invitations. Some identified overly complex (Q3), ‘clumsy’ sentences, with one participant, who was struggling to process a complex sentence, commenting:

(P13): It looks like a sentence that, if you were reading it out, you might trip over your words.

Summarising the consequences of ‘clumsy’ sentences another participant felt the researcher may ‘lose’ the potential participants (Q4). Participants in another focus group highlighted that poorly constructed sentences might make them mistrust the research team.

Participants wanted sentences that were ‘short’, ‘punchy’, ‘succinct’, ‘direct’ and ‘to the point*’,* identifying any aspects of invitations that they felt were written in this way. They suggested strategies to help researchers with this: one group felt that patients should be involved in reviewing COS invitations before recruitment begins; another participant described how the organisation she worked for used Artificial Intelligence technology to help ensure concise writing for the public. Several participants commented positively on the use of simple questions as sub-sections to structure the invitation such as ‘How can I help?’ Others highlighted how bullets helped to structure the information.

#### Understanding what the COS study was about

Most of the sampled invitations did not use the term COS, choosing instead to briefly state the aim of the study in a single sentence (referred to as ‘briefer explanations’ below). One invitation (an email) described what a COS was and its purpose in three sentences (referred to as a longer explanation below). This longer explanation was based largely on text from the COMET Initiative’s Plain Language Summary entitled ‘What is Core Outcome Set’ (www.comet-initiative.org/Resources/PlainLanguage), which was co-produced with patients and the public. The main difference between the text used in the email invitation explored in the GRIS study and the COMET Initiative’s explanation was that COMET’s included examples of what an outcome is.

Some participants seemed to quite like the longer COS explanation and appeared to process it in detail. They surmised that a COS was about standardisation (Q5), which seemed easier for them to understand than the detailed explanation. However, most participants appeared to have a confused or fragile understanding of the purpose of a COS study and what would be involved for participants (regardless of whether a brief or longer explanation was used). Many appeared frustrated and occasionally irritated by the COS descriptions provided:

(P14): The core outcome set is …. (reading invitation aloud). I mean that’s just gobbledegook!

Participants often attempted to make sense of the purpose of a COS from these descriptions, or what the study was about, however many struggled to do so (Q6 &7).

Numerous participants found the longer COS explanation particularly challenging, implying that it was overwhelming and describing it as a “big chunk” (P24) and a “dump” (P27) of information, while others talked about having to re-read these sentences (Q8). Several felt any explanation of the COS study purpose was unnecessary (Q9).

#### Understanding what a participant might be expected to do

Most focus groups discussed what might be involved in COS participation from a practical perspective. Several participants felt invitations gave insufficient information about the time involved, proposing that some indication of this should be provided, particularly when the target audience have a health condition (Q10). One group commented that the lack of information on what participation involved would deter them from taking part.

There were multiple pieces of information in the invitations that left participants confused about what taking part in a COS study would involve. One invitation mentioned that four stakeholder groups would participate in the COS—this confused a participant who was unclear how the study would be conducted with four groups. Another invitation mentioned a two-round study, which confused several participants and concerned another:

P30: It’s like if you do the first round but then you’ve missed the second round, is your opinion disqualified and have you wasted time with the first round.

Vague or unclear eligibility criteria were mentioned by some participants who were unclear whether a participant needed to meet one or all of the eligibility criteria (Q11). One participant summarised the basic information they would like to see in an invitation:

P31: If it says what it is, how you can help, how long it’s going to take you, what’s involved, and whether you're eligible. I feel like that’s everything you need to know whether you want to go ahead with it?

#### Information overload

While participants pointed to a lack of information and confusion about the practical aspects of participation, they also expressed concerns about the volume of other information in the invitations. They preferred invitations that were quick and easy to process. Several participants also made comments suggesting that they, or other people may skim through the invitation: “sometimes you just skip through, you just read the bits you want to read” (P11). The longer email (which was similar in length to those used in many COS studies) triggered considerable discussion with several participants describing the length as disengaging: “TLDR, - Too Long, Didn’t Read” (P19). While there were some exceptions, participants largely appeared to prefer invitations that were simple, calling for “basic information” (P7). One participant commented that a text-heavy invitation would mean “quantity over quality” (P4), later stating: “If I got sent that, I wouldn’t be reading it” (P4). One group, reviewing a brief social media post, responded positively to its brevity (Q12). Another participant highlighted how the volume of detail in the longer email invitation obscured key information in its midst, potentially disengaging a potential participant (Q13).

Several comments made by participants indicated that layering of information is important, that is, providing minimum information at the invitation stage, supported with more information later (Q14). Some participants were confused as to why some of the information in the invitation was not just in the participant information sheets, for example information on the purpose of COS (echoing points made about understanding the COS). They also called for duplication of information to be avoided or minimised.

### Safety

#### Trust

Given that researchers often rely heavily on online and digital methods of recruitment, we were surprised by the depth of scepticism, fear of online crime and data misuse that participants expressed when discussing the invitations. Most invitations that participants reviewed included a link for potential COS study participants to click on, either for further information or to register for the study. Several expressed concerns that the invitation may be a scam (Q15), using words like “wary” (P31), “sceptical” (P21) and “cynical” (P13) to describe their reactions. Some were concerned that their data would be sold or that the researchers may try to sell them something:

P9: One thing that goes through your mind when you get anything like this through email, you think are the doctor’s surgery selling off our information to private companies to get funding for the research.

Another participant was concerned about a disclaimer, which they perceived as suggesting that the organisation had some “controversial views” (P13).

Several participants described how they would attempt to ascertain the authenticity of the invitations, with some highlighting the importance of researchers including contact details so recipients have the option to authenticate the invitation themselves (Q16). A participant who had previously been a victim of a scam described how she meticulously verified requests to click on links by checking things like the email address and searching for the organisation on the internet (Q17).

#### Source and method of receiving invitations

The source and method of receiving the invitation appeared important to participants for verifying invitations. Some spoke of being reassured by the logos from health organisations, universities and patient organisations, which they felt reinforced the authenticity of the study. However, others were more cynical and voiced concerns about whether a logo was genuine, for example:

P31: I'm very wary about being scammed…… Even though it’s got a university logo, that doesn't really mean anything to me. Like it doesn't mean it’s legit… So, yes, I probably wouldn't click on it.

Regarding invitations from a patient organisation, some participants perceived that they could trust that the invitation was genuine because they were familiar with the organisation (Q18). Nevertheless, a few participants doubted the authenticity of an invitation from a patient organisation, possibly because they were unfamiliar with it. Several groups noticed that the opening sentence in one invitation referred to the study’s acronym without providing a statement to first introduce the study, or the research team running it. Some participants appeared unnerved by this, stating that the name of the research study was completely new to them (Q19).

A photograph of a researcher appeared on two of the invitations. The researcher in one of the photographs was wearing a white coat which one of the participants suggested added credibility. This contrasted with the same group’s comments about a less formal photograph of a researcher in another invitation, which they felt was less professional.

Some participants discussed alternative ways of receiving invitations that might foster trust, including hard copy invitations sent by post, information sent through the National Health Service (NHS) app or posters in clinical settings:

P31 Because if it’s in a clinic, I feel like, you can’t really go up and just stick random… they’re there for a reason. I feel like it’s more of a professional place. But to get an email with a link, I feel that email could come from anywhere

One participant talked about the process of getting involved in the current study, whereby they had met face-to-face with the researcher as part of the recruitment process. The participant indicated that developing trust in researchers through face-to-face interaction was important to patients.

#### Privacy, data handling and use

Not only were many participants concerned with the authenticity of COS studies, but many were also concerned about privacy, data handling and data use. Some questioned how the person sending an email would have accessed recipients’ contact details. Another worried about their data being passed to third parties without their consent (Q20).

Groups discussed the term ‘anonymous electronic survey’. Several participants talked positively about anonymity, but some felt that anonymity was confusing and potentially contradictory:

P7: But you can’t do an anonymous electronic survey. Of course, it’s not, is it? They’ve got your details as soon as you do it.

Some groups discussed the storage and confidentiality of data, with participants in one group voicing concerns about data being held indefinitely, despite reassurances from the facilitator that researchers were required to delete participants’ personal data.

### Feeling valued and respected

Participants were encouraged to consider whether the invitations made them feel valued and indeed this was found to be an important motivator for many participants. Several liked statements about the importance of their opinion, and that researchers wanted to hear from them, or that the COS study could not be done without their input (Q21). In their suggestions of how to improve COS invitations, numerous participants emphasised the importance of a message conveying the value that the patient brings (Q22). One participant emphasised how the use of the pronouns ‘you’ and ‘your’ helped to convey the importance of the patient’s opinions:

P22: The other thing about it is, “We’re interested in your opinions, we want to hear from you, please complete the survey.” It’s a very positive thing and you know that they do really want to know what you think and not what somebody else thinks (the words in bold were verbally emphasised by the participant).

Some researchers used phrases like ‘take part in our international study’ in their invitations, perhaps as a way of reinforcing the importance of the research. When asked about these statements, only one participant could see anything positive in such messages, reflecting on a potential family link with a condition. The majority, however, indicated that it made them feel that their contribution would be relatively insignificant (Q23 & 24).

Language that suggested superiority or hierarchy raised concerns among participants. One invitation mentioned that potential participants could contact the doctor’s secretary regarding the invitation, perhaps as a way of offering a more trusted way of communicating with the research team. However, this made some participants feel that they were being relegated to communicate with an intermediary:

P18: It’s almost like, I've told someone to send the letters, so don’t bother me.

Similarly, in a list of invitees on another invitation, patients were the last group mentioned in a list that included several different professions. One group picked up on this, concerned “we’re the last people” (P14).

### Engagement

#### What motivates and deters people?

Many aspects of the invitations were discussed that could potentially motivate or deter people from wanting to participate in COS research. Some of these have been touched on in the Understanding section above but in this section, we focus on which messages participants found motivating, demotivating or a mixture of the two.

#### Motivational

##### Learning

A few participants talked about the potential educational benefits of taking part in research. One participant spoke about possibly learning more about their condition. Another group talked about the importance of researchers feeding back a study’s results to the participants. One participant later reminisced about a research study that the group had previously taken part in, and no feedback had been provided, leaving them feeling “a little bit cheated” (P9).

##### Altruism

Not unexpectedly, several participants referred to altruism as a key motivational message. A few participants suggested that phrases about helping with research might be engaging. However, most participants seemed to prefer messages that referred to improving treatments or lives, rather than improving research (Q25 & 26). One group commented that a recruitment message about ‘improving research into the treatment of ….’ might be more impactful than ‘improving research’. This suggests that improving research is too abstract a concept, whereas ‘improving treatments’ is seen as more tangible, relatable and significant.

### Both motivational and deterring

Several aspects of the invitations were discussed that could both encourage and discourage participation including expertise, use of questions, reinforcing personal relevance and similarity, financial incentives, rarity of the research opportunity, invitational tone and language used, information about what is involved in the research and design features.

#### Expertise

We had anticipated that messages designed to make patients feel valued by acknowledging their expertise, such as ‘you can help by providing expert information about xxxxx (health condition)’ would be viewed as motivating by participants. Indeed, a few responded positively to such messages (Q27). However, many more participants responded negatively to this type of message. They were concerned about why patients and the public were being asked to be involved and why researchers could not solve the problem themselves. These participants felt that the public should not be considered experts and that such statements may induce a kind of imposter syndrome in potential participants (Q28 & 29). Instead, participants suggested referring to the person’s knowledge or experience, rather than their expertise (Q30). One participant however warned against using the term ‘lived experience’ considering this to be “jargon” (P13).

#### Use of questions

In the Understanding section we noted that the use of questions helped to structure the text for some participants. The importance of questions at the beginning of the invitation to motivate potential participants was also highlighted by several participants. One group, however, warned against questions asking if potential COS participants wanted their opinions to be heard, as they thought that patients that were happy with their care may decide not to take part (Q31). While questions do appear to be useful, consideration must be given to whether the question could potentially disengage some participants.

#### Reinforcing personal relevance and similarity

Several participants indicated that personal relevance and similarity with those mentioned in the invitation may be motivational, however some messages that indicated similarity were perceived negatively. There was almost complete agreement that a statement such as ‘patients have been involved in designing the study’ would be viewed positively by patients and the public, as it could make them feel the research is more pertinent (Q32). Some participants also commented that messages about the involvement of patients made the research appear more patient driven and the researcher appear more ’open-minded’ and willing to listen to patients’ perspectives (Q33).

One group talked about the potential relevance and importance of a patient organisation promoting the COS research, suggesting “you know they’re advocating for you and working for you and can help people” (P23). Another indicated that if an invitation came from such an organisation, they would feel it was from “ordinary people” (P2) and that this was motivating. Several participants described other ways that creating similarity with the potential participant could be motivational, for example if the researcher added messages conveying their personal interest in the COS study or if a message from a patient with the condition was included in the invitation, reinforcing the study’s importance.

#### Financial

One of the invitations offered research participants the chance to enter a prize draw to receive some vouchers. Some participants responded positively to this message and one group discussed the importance of financial incentives as recognition for the time the participants would be giving. However, the way the voucher draw was presented initially confused several participants, some of whom misread the information thinking that all participants would receive the voucher. One participant seemed concerned that by offering a prize draw rather than offering all participants a voucher, the incentive seemed a little disingenuous. Others felt that the offer of money would deter them, with one participant wishing that the money was spent on the research study, not on them as a participant. Participants were also concerned that by offering an incentive, the survey findings might disproportionately reflect the views of people who respond for financial reasons (Q34).

#### Tone of the invitation

The tone of the invitation was important to participants. One email, which was addressed ‘To the parent or guardian of ….’, was liked by one group, with a participant describing how it felt reassuringly familiar to them:

P21: I think it works. It’s the format we’d get letters home.

However, participants in several other focus groups disliked the tone of this invitation, describing it as “sterile” (P11), “bland” (P13), “matter of fact” (P14) and lacking “enthusiasm” (P14):

A member of another focus group advised that COS developers should aim to convey passion for the research and write invitations “like they were interested” (P6). Similarly, some participants wanted invitations to strike an emotional chord with them. The importance of a courteous tone was also emphasised when the language in one invitation was highlighted as “not so demanding, it’s inviting” (P10). Another participant highlighted the importance of putting *“*the reader’s point of view before the request” (P9) as a way of emphasising the value of the patient’s contribution.

Some participants raised concerns about the insensitivity of language in some invitations. One invitation was for a public health COS about healthy lifestyles in children and was aimed at parents. Several participants expressed concerns about this invitation, describing it as “a bit judgmental” (P20), or “you’re being told you’re a bad parent” (P21). Another social media post contained quite stark medical terminology for a condition affecting an intimate part of the body, which one participant described as downplaying the seriousness of the condition. These comments emphasise the importance of ensuring appropriately sensitive language and instructions in a COS invitation.

Clearly the tone of the invitation was important to many participants who inferred that the invitations needed to be respectful, sensitive, personal, egalitarian and enthusiastic.

#### What’s involved?

Despite prompting focus group participants to reflect on what they thought COS participation might practically involve and how this might influence their motivation to take part in a COS study, with the exception of the time involved, participants made little mention of this. Only one participant noted that the information about what was involved was potentially reassuring and motivational because: “It says that you’re not having to put too much time into it” (P14).

Two groups discussed the two rounds of the Delphi survey that were referred to in one invitation, which appeared to disengage them due to the level of commitment (Q35). One group discussed the limits of online participation, and pointed to the potential benefits of face-to-face participation for engagement:

(P13): It also means you’re not getting any refreshments with it being online – that’s the one thing that is really lacking, you know ‘Come for a brew and biscuits [laughs].

#### Design features

The design features of the invitations were a key focus of discussion among participants, emphasising both their potential impact on engagement and the care that needs to be taken with the visual design of invitations. Several participants said they liked the use of captivating images (Q36). An important issue raised by some groups however, related to incongruences in some of the images and logos used, which they found confusing and could, therefore, conceivably impact engagement. One of the invitations was targeting parents of children under 5 years; however, one group noted that the image used in the invitation appeared to be an older child. The use of a seasonal looking logo in another invitation was confusing to some groups, indeed some participants felt it would result in people not reading the invitation if that season had passed (Q37).

A university logo used in one invitation included the names of geographic locations of linked campuses internationally. This confused some, with one participant thinking that participation might involve having to travel to one of the countries mentioned. An invitation that included a multi-colour image of a world map confused participants as there was no key to the colours used in the image (Q38).

As previously described in the Safety section, two invitations included photographs of the researcher carrying out the study. Some groups commented on the potential positive impact of such a photograph, some describing how a photograph helped to convey that there is a “real person” (P14) behind the invitation or that it might ‘humanise’ the study. However, some participants were indifferent to the inclusion of the photograph. One found it hard to work out why a researcher would include their photograph, considering it “vain” (P12). Both the photographs were of a white woman which one group felt might influence who decided to participate.

Participants emphasised that research teams needed to be cautious when selecting images or designing logos, so that there is a clear relationship between the image/logo and the research study. As one participant said:

P17: The picture doesn’'t really link to anything to do with a xxxx (condition)

Formatting and presentation issues were highlighted by several participants, however there were differences in what people liked. While some of these preferences may relate to accessibility issues, others may reflect personal taste. For example, some liked certain colours and font sizes while others did not.

### Deterring

#### Lack of incentive

Participants talked about incentives in terms of the potential benefits of research to them or the wider patient community. Some participants appeared frustrated about the lack of clear benefits for COS participants in some of the invitations (Q39), aside from just helping researchers. Another participant highlighted how irrelevant the potential benefit of improving outcome measurement seemed to them.

P20: With something like xxxxxx (health condition), if you’re doing research, you need to convince the people who have it that what you’re researching is something that will be of actual benefit rather than, we’re just finding a better scoring system.

Similarly, a participant in a different group highlighted a message in one invitation, about helping the PhD student, which failed to incentivise her. She noted that “it has to benefit you as much as it does them*”*. (P14). Another participant described wanting something quite tangible from participation:

P13: if it was a cancer project, that you get involved in it, and that will increase the likelihood that the number of people who die from cancer will be reduced…, but …. what is the piece of research going to prove in the end?

#### Rarity

Messages about research being a rare opportunity predominantly generated a negative response, with some participants implying such messages could feel like blatant advertising (Q40). Additionally, participants were quite aware of the manipulative intent of such messages. One participant felt the rarity message was off-putting as it suggested some kind of finality of research in the clinical area:

P9: It makes it feel as though it’s a one-off. It should be ongoing, shouldn’t it? Research should be ongoing, not rare.

### Accessibility

Invitations generated numerous accessibility concerns among participants, including reading accessibility (both visual and literacy); linguistic challenges (where English was not the first language); issues related to hearing; and digital exclusion, practical accessibility.

#### Reading accessibility

Both visual and literacy issues were described by participants. One noted that potential COS participants who cannot read would be excluded from participating in a COS study. Another group noted that one of the study invitations mentioned that a video was available with additional study information. The group seemed excited about this idea of alternative media, describing how they found videos a helpful alternative to additional written information about the study: “you can get a load of information into a video” (P14). The colour and size of text in the invitations were a concern for some participants, particularly when considering the needs of older people and those with conditions such as dyslexia.

#### Linguistic exclusion

Several participants noted the potential for linguistic exclusion, with one group explaining that the already complex scientific language in the invitations would be even harder to understand for people whose first language was not English:

P16: … if you think all the words that we're having to learn and understand, if English wasn't even your first language-

#### Sensory accessibility

One group pointed out that one of the invitations was targeted at older people who may have hearing problems and that the invitation offered a telephone number for queries. Another participant explained that older participants may prefer to email the COS team due to hearing difficulties (although this will not be feasible for those without access to email or who lack confidence emailing).

#### Digital exclusion

Some participants liked the fact that the survey was available electronically, while others described digital exclusion challenges presented with trying to access the COS survey or register their interest online. For example, when hashtags were discussed in relation to a social media post, a deflated looking participant said “*…* they’re making things harder for everyone”! (P5). Another participant in the same focus group described being unable to access the digital world without their daughter’s involvement:

P4: About going on the link and that, me, I’m from the old school see and without my daughter’s help I wouldn’t even know how to go on the, how to switch it on, you know, what to do, you know. It’s all different these days.

Participants noted that not everyone has access to laptops, mobile phones, internet, email, etc. Another stated that documents, like the ones they were reviewing, rarely tell potential participants where they can go to use a computer. Quick Response (QR) codes were discussed and while some more internet familiar participants liked them, others appeared somewhat intimidated by them: “I’d look at it and go, more tech” (P20). A participant in another group pointed out that if a potential COS participant had seen an advert for a research study in a waiting area and wanted to write down the web address, some of these were long and liable to be written down incorrectly.

#### Practical accessibility

While accessibility issues were identified with online surveys some participants liked the fact that surveys were available online, particularly for people with health conditions and caring responsibilities (Q41). One group discussed the method of receiving invitations, with different preferences for emails and letters, indicating one size does not fit all in terms of survey delivery. Some groups, particularly those more familiar with technology, felt the process of accessing the survey in some of the invitations was needlessly complicated and time-consuming, preferring QR codes. Another participant suggested the need for easier ways of recording the contact details for the COS study team, such as pulldown tabs at the bottom of the flyer. One of the invitations gave participants the option of responding to the invitation via two different people, which caused confusion for one group:

P13: What I’d be worried about is, is one better to contact than the other?

## Discussion

If clinical research is to translate into real improvements in patients’ lives, patient important outcomes must be measured and reported. Patients are therefore key stakeholders in COS development, and it is important that COS studies are informed by diverse patient perspectives. Our study aimed to ascertain how diverse groups of patients and the public perceived written invitations for COS studies in terms of how understandable and engaging they are and whether they make participants feel valued. Our findings suggest that invitations contain too much information and are leading to confusion among potential participants about the purpose of COS studies and the terminology used. In a context in which the public are continually advised about the risks of cyber-crime, and research teams are often unknown to participants, concerns about safety and trust were key issues, especially regarding online safety and data management. Study participants suggested ways that invitations could be redesigned to be more reassuring for potential participants. In terms of engagement, participants wanted invitations which conveyed that the research had a meaningful purpose, helped participants to feel valued, that their contributions were significant and feel connected with others they could relate to. A further key theme was around accessibility, including considerations about digital and reading literacy and linguistic challenges in how invitations are designed and disseminated.

Despite our efforts to sample invitations that we considered reasonably clear, participants experienced numerous challenges in understanding the invitations. The vocabulary and language in the invitations were problematic for many participants, both in terms of jargon and sentence construction. Participants called for brief, clear sentences and use of questions to grab and maintain attention, indicating a preference for information that can be rapidly processed and invitations that are less cognitively demanding. Incomplete or confusing information required participants to give deeper consideration to what was being asked of them, which some participants seemed unwilling or unable to do.

The importance of using plain language and avoiding overly technical language was highlighted by participants. While this is old news in health research,^30–32^ it is important to highlight given the numerous resources available to support researchers to write in plain language (for example, Plain English Campaign, Digital.gov, Cochrane). The persistence of this problem in relation to COS invitations suggests a need for co-produced and piloted COS specific glossaries. Further research is also needed to understand barriers faced by COS researchers in accessing or using plain language guidance.

Study titles presented as acronyms were particularly problematic for participants. Acronyms tend to be used to simplify long study titles^33^ to create branding and aid recall, their use proliferating in studies such as clinical trials.^34 35^ However, unfamiliar study acronyms require decoding which adds to the cognitive load and demands more conscious effort. Several of our study participants became distracted and confused by such study titles, wondering why they were used and even describing them as risking losing the potential participant before they read on. Some participants called instead for a simple initial sentence in the invitation, such as ‘take part in our headache survey’, which likely requires less cognitive effort. While research has explored acronyms from the perspective of their quality^35^ and potential coerciveness,^34 36^ research is needed to explore at a more fundamental level how meaningful study title acronyms are for the public. Research is also needed to explore alternative ways of entitling research invitations or introducing a study in a brief statement.

Invitations with large amounts of information or with visually congested information also concerned our participants, with several calling for minimal, uncrowded information focused on the practicalities and potential benefits of taking part. This echoes concerns about the length of other participant facing resources in health research.^37^ Research suggests that documents perceived as complex or long may affect trust^38 39^ and may make participants think that participating in a COS study would be difficult.^40^ The detailed description of COS was particularly cognitively challenging. It is questionable whether a full explanation of COS is needed at the invitation stage. While co-producing research invitations with patients and the public may help to address some of the understanding issues our participants experienced, our findings reinforce the importance of also piloting invitations with the public who are not familiar with research.

We did not anticipate the safety issues that our study identified. Fear of cyber-crime was an issue raised by numerous participants as deterring prospective COS participants from clicking on the links embedded in invitations and using QR codes. This fear, which also led to distrust in organisational logos, is not without justification. Around 18% of the UK population reported being a victim of cyber-crime between 2023–2024, and these figures are likely an underestimate due to underreporting.^41^ Sugraphobia (fear of being duped)^42^ impacts internet use, especially among people aged over 50^43^ and it is conceivable that a request to click on a link may be becoming a trigger or heuristic that immediately deters potential participants.^44 45^ This is a major challenge for COS developers (and other researchers), who often opt for online recruitment and survey participation.^13^ Shifting recruitment to spaces the public trust, such as flyers in GP practices, face-to-face recruitment in clinic settings or communications from patient organisations may offer a solution. Indeed Fish^46^ reported higher retention rates among patients participating in Delphi surveys when they had been recruited face-to-face (compared with remote recruitment approaches). However, such recruitment methods will likely present logistical and financial challenges for COS developers.

Cialdini’s principles of persuasion^27^ developed in the field of psychology and marketing, were a useful framework for exploring factors that influence engagement and interest in COS invitations. Cialdini identified reciprocity as a key principle of persuasion, receiving something in return for your actions. Participants in our study generally liked to feel that their contributions would be valuable and that their opinions mattered, suggesting such social validation is a key message to convey in COS study invitations. Similarly, altruism was mentioned by several participants. Research suggests that being altruistic for some may be self-focussed, for example, benefitting from ‘the joy of giving’^47^ or being rewarded with a sense of happiness.^48^ Interestingly, participants we spoke to seemed more concerned with messages about improving treatment than research, suggesting they were more motivated by the idea of helping the treatment of others and less motivated by helping researchers or research processes.

Messages from a patient that the study is important to them, COS study endorsement from a patient organisation or learning that patients have been involved in designing the study were seen as important by many of our focus group participants, reflecting both Cialdini’s principles of social proof and liking. The finding that hearing about patient and public involvement (PPI) might positively influence potential research participants’ views about a study is consistent with findings from Neale *et al*^49^ who explored the views of people with opioid addiction about pharmaceutical clinical trial participation. Hudson *et al*,^37^ however, found PPI was poorly understood by patients and the public and that the concept can be interpreted to mean patients and the public are leading the research, resulting in concerns about credibility. Care should therefore be taken in explaining PPI.

We anticipated the statement mentioning that a study is international might link with Cialdini’s principle of unity, evoking a sense of belonging to an international group of patient participants. However, we were surprised to find that several participants regarded such statements as minimising their contributions, suggesting that the impacts of international participation are perceived as diffuse and intangible. Related to the principle of liking, participants also called for invitations to convey the researcher’s enthusiasm for the study, rather than a sterile tone.

Statements implying scarcity (such as ‘this is rare chance to take part in research’) is another of Cialdini’s principles but these were perceived negatively by our participants who appeared wise to such marketing approaches. However, our findings were consistent with Cialdini’s principle of authority with participants indicating, for example, that logos of respected institutions were perceived positively and a preference for professional looking photographs of researchers, although sugaphobia limited respect for logos in several cases (as these might be easily reproduced by scammers). The finding that many focus group participants preferred to not be referred to as experts also links to the authority principle, with comments suggesting deference to researchers or doctors as experts and providing insight into how participants perceived existing hierarchies and power dynamics.

We anticipated many of our findings on accessibility issues which indicate that a ‘one size’ approach of a single invitation for all may not serve populations who, for example, do not use computers, do not speak English or have learning difficulties such as dyslexia. Our participants called for invitations to be made available as hard copy to overcome some of these challenges and for invitations to be translated into different languages or designed around the needs of people with dyslexia. While pragmatic or financial factors may limit such steps, COS developers co-producing their studies with patients and the public may benefit from available guidance (for example, guidance on dyslexia and general guidance on accessibility) and from considering accessibility issues at the design stage of their studies.

### Strengths and weaknesses of the study

The key strength of this focus group study was that it was conducted with a diverse group of participants, many of whom were unfamiliar with the world of research. Several participants were from deprived communities who are continually underrepresented in research. We also incorporated real examples of previously used COS study invitations in our focus groups, enhancing the credibility of our findings.

There were however several limitations. Although we had some ethnic minority representation it was not extensive, limited to younger participants and our research was conducted in English, potentially excluding wider insights from those with diverse cultural backgrounds. Similarly, the study was conducted in the UK, and we cannot assume the findings are more widely transferable. We recognise that further research is needed to explore the perspectives of wider ethnic groups and international patient perspectives. The documents reviewed by participants in our study were sampled as they appeared clearer in their messaging than others. Our findings may therefore minimise the challenges potential COS participants experience. A further limitation is that while the study asked the participants to consider sampled invitations, this is different to reading an invitation as a potential participant in a real COS study. While participants provided insights into the lens that public COS participants might use when they read a COS study invitation, away from a focus group or interview perceptions might be different. Additionally, our participants were people who had been happy to take part in our study, and their perceptions regarding research may be more positive than the wider public.

## Conclusion

Patients are key stakeholders in COS development, so COS developers need guidance on how best to recruit a diverse patient population into their studies. Our study has explored the very first communication that a potential COS participant may read about a COS study, the research invitation. Our findings identify a wide range of factors that need to be considered when designing such invitations. This study was conducted as part of the wider Good Research Invitation Study. As a next step, we took our findings to an expert consensus meeting with patients, COS researchers, ethics committee members and experts in science communication, marketing and diversity to agree guidance for future COS research teams, which is reported separately.^24^

## Supplementary material

10.1136/bmjopen-2025-111016 online supplemental appendix 1

10.1136/bmjopen-2025-111016 online supplemental appendix 2

10.1136/bmjopen-2025-111016 online supplemental appendix 3

10.1136/bmjopen-2025-111016 online supplemental appendix 4

## Data Availability

No data are available.
