# Supplementary material for: Do patients and the public understand and feel engaged and valued after reading outcome set study invitations? A UK qualitative study
Source: BMJ Open. 2026 Aug 18;16(8):e111016. doi: 10.1136/bmjopen-2025-111016 (PMC13504493; doi:10.1136/bmjopen-2025-111016)
Supplement: Supplementary file 1 [file bmjopen-2025-111016.reviewer_comments.pdf]

PEER REVIEW HISTORY

BMJ Open publishes all reviews undertaken for accepted manuscripts. Reviewers are asked to complete a checklist review form and are provided with free text boxes to elaborate on their assessment. These free text comments are reproduced below.

ARTICLE DETAILS

Title (Provisional)

Do patients and the public understand and feel engaged and valued after reading outcome set study invitations? A UK qualitative study.

Authors

Barrington, Heather; Craven, Rebecca; Young, Bridget; Williamson, Paula

---

VERSION 1 - REVIEW

**Reviewer**	**1**
**Name**	**Sun, Virginia**
**Affiliation**	**Division of Nursing Research and Education, Department of Population Sciences, City of Hope**
**Date**	**19-Jan-2026**
**COI**	**none**

Paper is well written and clear.

**Reviewer**	**2**
**Name**	**Probst, Pascal**
**Affiliation**	**University of Heidelberg, Department of General, Visceral and Transplantation Surgery**
**Date**	**17-Feb-2026**
**COI**	**none**

Barrington et al. present a qualitative study on patient and public perspectives regarding invitations to participate in core outcome set research. The rationale for conducting this work is clearly described, the methods are appropriate, the results are presented in a clear and accessible way, and the conclusions are measured and balanced.

Studies of this kind are still relatively rare and, in my view, worth publishing. I found the paper genuinely insightful, particularly in how it illuminates patient perspectives on research

invitations, which is highly relevant for improving recruitment approaches in core outcome set studies.

The key limitation is transparently acknowledged by the authors: participants in this qualitative study were willing research participants and may therefore hold more positive views about research invitations than the wider public. This concern is valid. Nonetheless, within the constraints of qualitative research, the findings represent the best available evidence on this question and provide practical, patient-informed guidance for designing clearer and more acceptable invitations.

**Reviewer**	**3**
**Name**	**Cristall, Nora**
**Affiliation**	**University of Calgary Cumming School of Medicine, Medicine**
**Date**	**17-Mar-2026**
**COI**	**none**

This is an important topic that you are advancing and contributing a vital patient voice.

Patient and public perspectives outcome set study Invitations: qualitative findings from the Good Research Invitation Study

Thank you for asking me to review this manuscript. I do not recommend moving forward with revisions. This manuscript is far too long at 9375 words.

Secondly and more importantly, the sample has limited ethnic representation outside of "White" older participants. All of the participants identifying as an ethnic minority also reported being under age 30. Further Focus Groups could assist with filling out other voices and would contribute substantially to this building body of work. Here are some additional comments.

1. The abstract could include more information on the value of patient input and co development of material. This paper should be readable as a standalone manuscript without necessarily having all of the background articles. The authors should reiterate the value for patients.
2. On page 3 The footnote is distracting, and this information can be included in the text. may be a better way to present
3. On Page 3in the Background Line 5 in the summary could be deleted. In this paragraph the word outcome is used too much without a clear definition. Tijs should be presented in plane language.
4. Page 5 there is no need to include the Principles of Persuasion diagram or point summary. This can be briefly referenced.

5. On Page 8 the design summary needs to be condensed.
6. On page 7 in The Results table I note the bias in representation to older white men and women. This is not a representative sample of patients or community members. The findings this far could be built upon by collecting more data.

Respectfully Submitted

Nora Cristall, MSW PhD,

VERSION 1 - AUTHOR RESPONSE

Response to reviewer's comments:

Dear Mr Aldcroft
We would like to thank you and the reviewers for your time and for the thoughtful and constructive comments on our manuscript. Please find below our responses to the comments:
Reviewer 1
No changes were required for this reviewer.

Reviewer 2
No changes were required by this reviewer

Reviewer 3
1. "This manuscript is far too long at 9375 words."

We recognise that this is a long article, however we feel its length reflects the richness of the data and the importance of giving justice to the participants' perspectives. We have seen similar length articles published by BMJ Open, for example a recent edition of BMJ Open included this article: https://bmjopen.bmj.com/content/bmjopen/16/3/e109458.full.pdf. We have however made some edits to address the reviewer's comment.

2. The sample has limited ethnic representation outside of "White" older participants. All of the participants identifying as an ethnic minority also reported being under age 30. Further Focus Groups could assist with filling out other voices and would contribute substantially to this building body of work.

Thank you for this important point. Whilst our participants were diverse in age, gender, socioeconomic status and level of education, we agree that ethnic representation was more limited, particularly in the older age range. We recognise that further ethnicity focused research is needed to adequately capture the breadth of cultural perspectives. While this was beyond the scope of our research (conducted within a time and resource limited PhD), we have now revised the manuscript to more clearly acknowledge this issue as a limitation. Specifically, we have expanded the "strengths and weaknesses of this study" section to reinforce the need for further research in this area and we have also highlighted this need in

the Article summary - Strengths and limitations section.

3. The abstract could include more information on the value of patient input and co-development of material.

We have now revised the abstract to the following (change is in bold): COS are developed with input from key decision makers, patients being a vital group to ensure COS relevance.

4. This paper should be readable as a standalone manuscript without necessarily having all of the background articles.

We have reviewed the manuscript in the light of this comment and feel the background articles referred to are necessary to justify our reasoning and methods and consider the findings in the light of previous research. If necessary, we could reduce the number of references.

5. The authors should reiterate the value for patients.

Thank you for this useful suggestion we have reiterated this at the beginning of the discussion, and we feel this has helped emphasise the importance of our study findings.

6. On page 3, the footnote is distracting, and this information can be included in the text. may be a better way to present.

We have now included this information in the text of the Introduction.

7. On Page 3 in the Background Line 5 in the summary could be deleted.

We are sorry but we are unclear which text the reviewer would like us to delete.

8. In this paragraph the word outcome is used too much without a clear definition. This should be presented in plain language.

We have added the following plain language definition of the word outcome “what is measured in a study to assess if an intervention works”.

9. Page 5 there is no need to include the Principles of Persuasion diagram or point summary. This can be briefly referenced.

We feel that some readers may value this information being to hand when reading the article but take the point that it may be too much information for the main article and therefore we have removed the summary from the main text of the manuscript and instead added this information as a new appendix (Appendix 2), referencing this in the main text.

10. On Page 8 the design summary needs to be condensed.

We have made some revisions to condense the Design summary and the rest of the Methods section (as described in point 1 above). However, in order to be compliant with the SRQR guidance we have retained information specified by this guidance.

11. On page 7 in The Results table I note the bias in representation to older white men and women. This is not a representative sample of patients or community members. The findings this far could be built upon by collecting more data.

This has been addressed in our comment on point 2 above.

VERSION 2 - REVIEW

**Reviewer**	**3**
**Name**	**Cristall, Nora**
**Affiliation**	**University of Calgary Cumming School of Medicine, Medicine**
**Date**	**01-Jul-2026**
**COI**	

Thaank you to the authors for taking the time to integrate suggestions. I feel this will make for a more readable adn interesting document.
