# Supplementary material for: Do patients and the public understand and feel engaged and valued after reading outcome set study invitations? A UK qualitative study
Source: BMJ Open. 2026 Aug 18;16(8):e111016. doi: 10.1136/bmjopen-2025-111016 (PMC13504493; doi:10.1136/bmjopen-2025-111016)
Supplement: Supplementary file 3 [file bmjopen-16-8-s002.docx]

**Appendix 2**

**Cialdini’s Principles of Persuasion and their potential relationship to study recruitment**

- **Reciprocation** – People feel obliged to return a favour or repay kindness (for example receiving a gift with a research invitation).
- **Liking** – People are more readily persuaded if there is some form of connection with others like themselves or if there is a positive emotion felt to the person sending the invitation.
- **Social proof** – People prefer doing something if they see others doing the same thing (such as hearing that others are taking part in the study).
- **Authority** – People trust those that they perceive to be experts (such as an invitation from an eminent university)
- **Scarcity** – People value something more they perceive it to be rare (such as a rare chance to take part in research).
- **Commitment and consistency** – People like to be consistent in things they say or do (hence pre-registering for a study may encourage consistency, as the person has already made a commitment).
- **Unity** – People prefer to agree to people that they share an identity with or feel a sense of belonging with (for example if an invitation is sent from a patient organisation that the person belongs to).
