# Supplementary material for: Do patients and the public understand and feel engaged and valued after reading outcome set study invitations? A UK qualitative study
Source: BMJ Open. 2026 Aug 18;16(8):e111016. doi: 10.1136/bmjopen-2025-111016 (PMC13504493; doi:10.1136/bmjopen-2025-111016)
Supplement: Supplementary file 4 [file bmjopen-16-8-s003.docx]

**Appendix 3 - Focus group topic guide**

**Note for ethics committee -** This document indicates the types of topics to be explored. However, it is not prescriptive and in keeping with good practice in qualitative research the types of topics and questions will be adapted to the needs of the participants and evolve over the course of data collection and analysis.

**Key points for reseracher**

- Consent form
- Audio recording
- Confirm duration of focus group / refreshment break
- Explanation of process (see ‘Reading invitations’ introduction below)
- Provide invitations, checklist for reviewing the invitations (Appendix 11.1) example webinar invitation (Appendix 11.2 [omitted for double-anonymized peer review]), post-it notes, stickers, highlighter pens, pen, paper, and space to read
- 6 sheets of paper on the wall headed ‘interested me’ ‘put me off’ ‘made me feel my contribution would be valued’, ‘didn’t make me feel I was a valued participant’ ‘helped me understand’ ‘confused me’ + an extra sheet for any other comments

**Introduction**

***Consent***

Take consent

***Ways of working***

Agree ways of working

***Reading invitations***

I. Researchers use invitations to ask people to take part in their research. We want to find out what makes a good research invitation from a public and patient perspective.
II. To help us find this out, we will ask you to read a few research invitations. We’ll read a couple at a time and then discuss them. We’ll stop for a refreshment break part way through.
III. When you read each invitation it’s important to understand that when people are invited to take part in a research study, they usually get both an invitiation AND a detailed information sheet about the study. The information sheet will explain the purpose of the research, what will happen if the person decides to participate, any ethical or data storage issues, that kind of thing. So the invitation is the very first thing the patient would read, before reading more detail about the study in the information sheet.
IV. When you read each invitation. I realise that you won’t have the condition that is being described, so I just want you to imagine that the invitation is relevant to you when you read it, so for example, imagine you are a person with that condition and you receive this invitation.
V. At first, when you’re reading each invitation, I’d like you to think, is this a good invitation? Is it average, or is it poor (just based on your gut instinct when you read it)? Please use these stickers to score how good you think they are. So green happy face for good, amber neutral face for average and red unhappy face for poor. There are no right or wrong answers here. Different things appeal to different people, and different people will understand different parts better than others, so we fully expect a wide range of views from the group.
VI. Then, as you’re going through each invitation I’d like to to use the checklist we’ve given you, when you’re reading (Appendix 11.1), I’d like you to think about any words, phrases or sentences in the invitations that:
    a. Help you understand
    b. Confused you – anything you didn’t understand, or you had to keep re-reading to make sure you’d understood it properly
    c. Made you interested in the invitation – so imagining you had been received the email or you’d seen the social media post – were there any parts of the invitation that made you interested in the study and that would make you want to take part
    d. Put you off – Any parts that made you think – ‘no chance’ that bit made me not want to take part
    e. Made you feel like your contribution would really be valued or that you would have a really important voice in this process
    f. Didn’t make you feel valued or that you would have a really important voice in this process

We’ll have a practice on this event invitation (Appendix 11.2) If you can first read the invitation, then put a sticker on it as to whether you think it’s good, average or poor as an invitation. We will then have a brief discussion picking out things you liked or didn’t like (using the checklist as a guide).

(Allow time to read the example).

**Main Discussion**

***1 - General***

1. Hold two invitations up, say the title and ask hands up for green / amber / red. For each invitation ask why red etc. (*researcher will encourage contrasting views to avoid group think*).

***2 - Understanding***

During the group discussion, write down difficult or easy-to-understand words. Remember different people often find different things easy or difficult to understand. So don’t worry if only you found something difficult or easy. We want to get a range of views.

2. You’ve read <insert number> research invitations today. What are your views on why researchers ask patients to participate in these studies? In your view, is it important for patients / the public to take part in COS studies? (If yes, what makes you think that?)
3. Do you think core outcome set research matters to patients? What makes you think that from the initiations what you’ve read?
4. Were any of the invitations easier to understand? If so, which one(s)?
5. Were there any particular words or phrases that helped you understand it?
6. Were any of the invitations confusing? If so, which one(s)?
7. Were there any words, phrases or sentences that made it difficult for you to understand.
8. What about the title/ email header?

**3 - Engaging**

9. Did any of the invitations make you feel you would want to take part? Which one? What was it about that invitation that might make you interested in taking part?
10. What words/phrases or sentences made you interested? Why?
11. What words or phrases put you off and why?
12. What about the title/email header?
4 **- Importance of the patient voice / feeling valued**

13. Did any of the invitations make you feel like you would really have a voice in this research? Which one? What made you feel like that?

14. Did any words, phrases or sentences that make you feel like that?

15. Did any of the invitations make you feel like your contribution would really be valued?

16. Did any words, phrases or sentences that make you feel like that?

17. What about the title/ email header?

**5 – Anything else?**

18. What were your thoughts on the presentation of the invite (was the text clear / well-spaced / any images helpful)?

19. What would be the three key things you would say are important for researchers to know when producing a social media / email / flyer invitation for a patient (ask this after discussing each batch).

6 **- Other potential messages**

20. Research study invitations need to be short and engaging. Bearing this in mind, here are some key messages that could potentially be used in a brief research invitation (point to sheets of paper on the walls with key messages on, which participants also have a copy of). We fully expect that people will like different messages for different personal reasons. Please could you now look at the messages and rate which messages would most make you want to take part write number 1 next tor the one the you like most, down to number 7 for the message you like least. Looking at your responses to each message, what did people like or dislike about this message? And what made you feel like that?:

**Example messages to be used**

a. 1000s of other patients have been involved in similar studies to this in the past
b. The University of xxxx is conducting this research
c. This research study is being conducted with the (examples) World Health Organisation / Cancer Research UK / The Royal College of Surgeons
d. This is a rare opportunity to give your views on ……….
e. A group of patients helped to design this research study
f. We (*the research team*) want to improve research into x condition …..
g. a testimonial (statement) from an important organisation/person saying why they think this study is important

**Closing focus group**

21. That’s the end of my questions. Is there anything else that you feel it would be important for researchers to know when they are designing their research invitations for studies like this?

22. Is there anything else you’d like to say?

Close focus group

**Appendix 11.1**

**Checklist for reviewing the invitations**

- Help you understand the invitation
- Confused you
- Made you interested in the invitation
- Put you off the invitation
- Made you feel like your contribution would really be valued

**Appendix 11.2 - Sample invitation for practice session**
