# Supplementary material for: Do patients and the public understand and feel engaged and valued after reading outcome set study invitations? A UK qualitative study
Source: BMJ Open. 2026 Aug 18;16(8):e111016. doi: 10.1136/bmjopen-2025-111016 (PMC13504493; doi:10.1136/bmjopen-2025-111016)
Supplement: Supplementary file 5 [file bmjopen-16-8-s004.docx]

**Appendix 4 – Additional quotations supporting the Good Research Invitation Study findings**

| **Main Theme** | **Sub theme** | **Quote** |
| --- | --- | --- |
| **Understanding** | **You've got to get the words right** | **Q1** - P15: "…I*f you put things in the abstract like the word outcome, that's the future, it's not a definite thing, people don't always understand that. So, you need things to be concrete*". |
|  |  | **Q2** - P20*: “If you start looking at business-speak, or medical- speak or whatever else, as soon as those things start to creep in, that is when you lose people”.* |
|  | **Sentence construction and presentation** | **Q3:** P24*: “They just use a very long sentence for a very short message”.* |
|  |  | **Q4** - P9*: “It's almost losing them before you’ve got to them”.* (referring to complex sentences) |
|  | **Understanding what the COS study was about** | **Q5** - P9*: “If they’d just put something like they want to standardise the core outcomes from various tests…. but because it was so detailed, so it doesn’t read easily”.* |
|  |  | **Q6** - P17*: “what it means is they’re going to do more research into why people get infections”.* |
|  |  | **Q7** - P9*: “They seem to be going to test the different medications different people are on and the outcome. But that’s what I read twice to understand it”.* |
|  |  | **Q8** - P23*: “I do understand it but with the amount of brain power, I would probably have to go back and read it at least two more times to really- it would make it hard for me to think about what they actually want, because all the time I’m like, wait, what do they want”?* |
|  |  | **Q9** - P3*: “Why is that even there?"* (Referring to COS description) *…Is that even important to be there”?* |
|  | **Understanding what a participant might expected to do** | **Q10** - P17*: “If they’ve got chronic conditions. And just managing their condition takes up a lot of time in your day. You’re thinking, well, is this a one-off thing? Is it something where you're going to want my continued commitment over a period of weeks or months? You just need to know the timeframes, how much time you're involved in”.* |
|  |  | **Q11 -** P14 (discussing eligibility criteria)*: “I don’t know, do you have to have all of them”? …..*  P13 *"And I would have thought it was all - I would assume it was all of them".*  P14 *"I was looking at it thinking it was just one of them”.* |
|  | **Information overload** | **Q12** - P8 (referring to a brief social media invitation)*: “I actually think I would do that one…. it’s short and to the point”.* |
|  |  | **Q13** - P29*: “I feel like you would have to read it all though because it’s only about halfway down the page they mention it’s an online survey. Like up until when you read that it seems like quite a big deal and then it’s just an online survey halfway down”.* |
|  |  | **Q14** - P23*: “You could just say to complete a survey … and then they could deal with the method of doing it once the people contact them”.* |
| **Safety** | **Trust** | **Q15** - P16*: “I just feel unsure of it and wonder… where it’s going to take me and there are so many scams about aren’t there?”* |
|  |  | **Q16** - P2*: “You’ve got the name of the university, you’d phone up, wouldn’t you? ... if you weren’t sure about it, that’s going to be spam".* |
|  |  | **Q17** - P24*: “I look at the logo, does it make me feel safe anymore that it’s really from the original sender? I check the email address and I Google, if it’s the right one I’ll get back to them”.* |
|  | **Source and method of receiving the invitations** | **Q18** - P24*: “I know this organisation; I know it’s not a scam or anything. The website is an original website. I’m happy to help them”.* |
|  |  | **Q19** - Interviewer: “*What would make you open an email to do with health research*”?  P7: “*I would have to know who it’s from. I don’t know who xxxx* (study name) *is*.” |
|  | **Privacy, data handling and use** | **Q20** - P1*: “Once you’ve got, they’ve got your details, are they going to pass it on to someone else?”* |
| **Feeling valued and respected** |  | **Q21** - P24*: “They want to use my thoughts, ideas and experience. I think that’s a nice one. I would just like to go for this study. They make my thoughts valued”.* |
|  |  | **Q22** - P17*: “Speak in such a way, like, whichever individual reads it, they feel like they're going to be heard. Because nobody wants to invest time in doing something if you think it's just a pure tick box exercise”.* |
|  |  | **Q23** - P14: *“I feel like a drop in an ocean… I don’t feel as though I’m important…. You must have loads of other people”.* |
|  |  | **Q24** - P19: *“It makes you feel like a really small cog in the thing, so, actually, I would feel insignificant more, I guess. For some people, it might appeal to their sense of importance”*. |
| **Engagement - Motivational** | **Altruism** | **Q25** - P2: “*Just as long as it, makes a difference or change in someone’s life*”. |
|  |  | **Q26** - P6: “*I’d feel I could be helpful in contributing to future treatments*”. |
| **Engagement - Both motivational and deterring** | **Expertise** | **Q27** - P31: “*Expertise again …. it's just a big word that makes you feel good, you know*”? |
|  |  | **Q28** - P26: “*I don’t like the expert because I’m not*”. |
|  |  | **Q29** - P11: “*You’d be worried about putting the right words in, expert*”. |
|  |  | **Q30** - P14: “*Experience sounds more like anybody can do it, ‘cause everybody has experienced something. Not everybody feels like an expert, do they?*” |
|  | **Use of questions** | **Q31** - P16:” *I wonder if sometimes when you ask a question like that, you only get the people who aren't happy….. Because if you’re happy*"…  P15: "*You wouldn’t be interested*”. |
|  | **Reinforcing personal relevance and similarity** | **Q32** - P17 “*I think people might think the questions might be more relevant to them*". |
|  |  | **Q33** - P12: “*Feel like I’d look at it more because there’s a bit more to it than some professionals sat in the office*”. |
|  | **Financial** | **Q34** - P23: “*Are you only going to get people who want the voucher? So, you’ll only get the answers of people who want a voucher*”. |
|  | **What's involved** | **Q35** - P28: “*It seems like a really long process”.*  *Interviewer: “Would that have put you off”?*  *P6: Most definitely. Yes*”. |
|  | **Design features** | **Q36** - P14: “*It’s quite eye-catching as well, from a distance*”. |
|  |  | **Q37** - P17: “*I think the more I look at this now, the more I think that if it wasn't xxxxxx (season), you wouldn't even look at it……*"  P18 "*Yes, that should have been taken down"*.  P17: "*You’d think that’s been up there a while*”. |
|  |  | **Q38** - P16: *“What does that mean? What's the relevance of that? Is that like xxx (health condition) population? Is it where the clinical trials are going on"?*  P17: *"Is it where people have responded from”?* |
| **Engagement - deterring** | **Lack of incentive** | **Q39** - P23: “*Why, what for, benefits? There is no information there at all, why would you do it? You know, who are you trying to help …. There’s nothing*”. |
|  | **Rarity** | **Q40** - P31: “*I’d feel like it's kind of trying to drag you in*”. |
| **Accessibility** | **Practical accessibility of the COS** | **Q41** - P22: “*I think the fact that it says to parents, it’s online when they’re very busy and can’t attend things is probably the biggest pull for me personally, because you can find half an hour or something to do something, whereas going somewhere with little ones is often quite difficult*”. |
